# In search of conceptual frameworks for relating brain activity to language function

**DOI:** 10.3389/fpsyg.2014.00716

**Published:** 2014-07-11

**Authors:** Mike A. Sharwood Smith

**Affiliations:** Moray House School of Education, University of EdinburghEdinburgh, UK

**Keywords:** multilingual, bilingualism, second language acquisition, second language processing, neurolinguistics, psycholinguistics, models, theoretical

The focus of the current topic is the analysis and interpretation of second language (L2) and multilingual data. Looking at data from speakers who have learned their additional languages after the mother tongue has become well established is of special interest. It advances our knowledge about how different language systems share space in the same mind, a question to be asked of any kind of multilingual at any age—and secondly it can tell us more about potential differences between early and later learned languages (Kim et al., 1997; Kovelman et al., 2008). More recent research points to brain areas activated in late learners of L2s becoming more and more like those of L1 acquirers as their proficiency advances (Green, 2003). At earlier stages of acquisition, adults may simply adopt compensatory strategies, for example recruiting new cognitive resources that have become available with increasing maturity to complete communicative tasks that are demanding either because, unlike very young children, they personally want or are compelled by interlocutors to communicate complex ideas, or because the requirements of a given experiment simply make the tasks demanding. This will therefore implicate regions of the brain that are much less involved in young language users and these may stay involved even where higher levels of L2 ability render them much less important or even unnecessary. In any case, to get the full picture we need ways of tracking this strategic activity, one aspect of which is the deployment of explicit processes, both those involving conscious awareness and those that may be raised to awareness but can also operate subconsciously but there will surely be processes that only operate subconsciously as well (Sharwood Smith and Truscott, 2011). These will affect not only the spontaneous uses of L1 and L2 but also performance on experimental tasks. Tracking brain activity with sophisticated apparatus is not enough of course: the data needs to be analyzed and for this we need very sophisticated theoretical frameworks to guide interpretation.

While research techniques such as brain imaging are gradually acquiring greater precision, helping to reveal much more about brain activity associated with linguistic processing, there still remain many problems interpreting results. This may not be an immediate problem in a given experiment because the research question may be suitably precise and focussed enough to guarantee an answer of sorts in the hope that answers to limited questions may gradually accumulate and provide the basis for wider explanations. In this way, for example, syntactic and semantic processing can be teased apart on the basis of participants' differing responses to examples of, respectively, syntactic and semantic anomaly which then allows researchers to identify separate neural signatures and provide support for particular accounts of the status of language vis-a-vis other types of cognition. Issues of interpretation become more evident when trying to put results into a wider explanatory context. One problem concerns the choice of which theory and which concepts and categories to import from a neighboring research domain. Another one, related to that, is locating conceptual models and frameworks in related domains (neurolinguistic, psycholinguistic, theoretical linguistic) that can be combined in such a way as to promise the best possible explanation.

Assuming the focus is on explaining language, and leaving aside sociolinguistic issues, if we get down to the basics, what do we have? 1.3 kilos (three pounds) of soft tissue and our current understanding of its functional architecture. Add to that theories about psychological function and, in many cases at least, entirely separate, well developed theories about linguistic structure. Each of these theories has, for very good reasons, its own conceptual framework and terminology, and its own favorite methods of investigation. For satisfactory explanations of how the brain stores and processes languages, we need somehow to coordinate findings in all these different disciplines. At the same time, it is not a straightforward job to bootstrap, for example, a Minimalist approach to explaining language structure to a model of human memory and make it into a real-time processing theory. This is true notwithstanding the obvious need, in the elaboration of theories of processing and development, for fine-grained accounts of linguistic structure. Standard generative linguistic approaches to language employ terms and concepts to explain abstract linguistic structure that are outside time and space. Without going into the details, these are notions like “move,” a structure-building operation changing the position of some item in a structure, “merge,” a combining operation, and “feature-checking,” the process whereby two associated items in a structure are assessed to see if they can be “licensed,” i.e., co-exist in their current position (in a particular manner determined by the theory). The use of such spatiotemporal metaphors, without any responsibility for explaining processing facts, is a highly effective device for describing structural relationships in syntax. At the same time these metaphors should not be translated straightforwardly into real-time, on-line processing terms. If they were thus interpreted, that would constitute a serious category error—confusing abstract theoretical linguistic concepts for ones that are custom designed to describe and explain events in real time. Either that, or it would constitute a new and different type of claim entirely, i.e., that the abstract concepts can do double duty and describe real-time events *as well.* Unless there is such a claim, working with linguistic theory means operating at a different level of description from *psycho*linguistic processing theory. Yet another level, distinct from both of those ones, seeks to provide *neuro*linguistic descriptions and explanations (Sabourin and Haverkort, 2003). Again, there is no guaranteed simple and straightforward translation of psychological explanations into neurofunctional ones either. In a psychological description, a working memory (WM) may be described as, say, a single module where its neural substrate is seen as being distributed over, say, three different systems, each located in different areas of the brain. If the model of memory is a modular one, the number of subsystems can still be different depending on whether the description is psychological or neurological.

Although functional models in psycholinguistics do not have to match their linguistic and neurolinguistic counterparts in any literal way, the need to interpret one into terms of the other does have to be acknowledged so that the search for, or development of compatible models across disciplines can proceed. While research continues sorting out the “easier” problems of data collection and analysis within each of these three disciplines, it is still useful to cast a critical eye on many of the basic assumptions being made and raise some questions about them. It is fair to point out that with the increasing sophistication of techniques that record brain activity, the problem of resolving the bigger questions will gradually become more tractable but only provided that suitable theoretical, compatible, well-founded models in companion domains can be identified so that the bigger questions can be formulated.

To take memory as a case in point, what is the relationship between on-line language processing, in this case by bilinguals (multilinguals) and the formation of new stable memories? How and where are the relevant memories formed? Should different types of memory be assumed as is often the case nowadays. If so, how many? Let us begin, say, with the initial registering of the acoustic stream: if we can accept that auditory-acoustic memories are formed in the primary auditory cortex, where and how is the subset of those memories that are identified as language-relevant processed further, thereby forming (some claim) separate types of memory? Is it legitimate to talk, for example, of a single separate “linguistic memory” system or are there in fact two separate types of memory involved, phonological and syntactic (Jackendoff, 2002; Truscott and Sharwood Smith, 2004; Sharwood Smith and Truscott, 2014)? Moving on to WM, is this part of a unified system serving all types of cognitive and perceptual activity or is WM also modular and domain-specific? If so, which modules and which domains are we talking about? And, during repeated on-line processing, when items that have appeared in (one or other instances of) WM have eventually become stable and established items in longer term memory (LTM), should we treat this acquisition process as resulting from the successful transition of the relevant items from one memory system (WM) into another one (LTM) or, alternatively, should we treat WM and LTM as part of a single memory system thereby characterizing acquisition as establishment of an enduring trace in LTM that then becomes increasingly accessible over time (Cowan, 2005; Baddeley, 2012)? It might not matter which option you choose for some purposes but if the models are going to be useful they may each have different empirical consequences when applied to the more complex questions of linguistic acquisition and performance. The plain fact is that models being used today still do not yet specify exactly how language systems are stored and used within one mind/brain. A much more detailed architecture is required to required to meet this requirement and explain how discourse/pragmatic, semantic, morphosyntactic and semantic features are stored and interact across a single or across multiple language systems (see, for example, proposals in Sharwood Smith and Truscott, 2014).

Connected with the decisions about which model of memory and storage to use is the question whether or not there is anything like a “language acquisition device” and if there is one, how does it work? What is its neural substrate? To take representational models of cognition, for example, some assign a special status to human language while others treat it as part of general cognition. The emergentist architecture proposed by O'Grady is an example of the latter (O'Grady, 2000). O'Grady explains language acquisition as cognitive development that is driven by the selection of ever more efficient processing operations to handle the input. One such operation seeks to minimize the burden on WM. Sharwood Smith and Truscott's account is similar at least in this one respect, denying the need for a language acquisition device, whereas Carroll's Autonomous Induction Model is different and posits a modular, failure-driven acquisition mechanism that is unique to language (Carroll, 2001, 2007; see discussion in Truscott and Sharwood Smith, 2004; Sharwood Smith et al., 2012; Sharwood Smith and Truscott, 2014). In any study of acquisition, it is fair to ask what background theoretical commitments the researchers are making and to what extent it is a matter of principled choice or just one of convenience, understandable as that might be.

My final example is the notion “representation.” If we set aside non-symbolic accounts, somewhere along the line we have to have a clear idea of how to treat representations at the different levels of description (and explanation) that we have been dealing with, ranging from simple ones like “word,” “syllable,” and “lexical item” to ones like “noun,” phonological and syntactic “features” and the whole gamut of theoretical categories that we wish to deploy in some form or other for experimental investigation and data analysis. Representations may be psychological constructs but they should have neural correlates. For example, Damasio's notion of “dispositional representation” meshes easily with the way linguistic or psychological representations are conceived. A dispositional representation is “a potential pattern of neuron activity in small ensembles of neurons and may be distributed over a number of different locations in the cortex, the precise locations depending on the type of representation and whether it is innate or acquired as a result of experience” (Damasio, 1994, pp. 102–105). This provides another illustration of how the neural equivalent of a psychological representation located in one particular place in a theoretical model can be a structure that is distributed across the neural system in different places. It also shows, incidentally, that you do not need to choose between symbolic representational accounts on the one hand and connectionist accounts based on (biological) neural networks on the other. Networks, representations and modular architectures can live peacefully together.

To some extent this short discussion is more a look into the future than a critique of past and present research. It is somewhat of a cliché to say research in this area needs to be conducted by teams from different research domains. To some extent this is already happening. My basic point is that the development of useful conceptual frameworks that can support such multidisciplinary research is still in its infancy. I have my own suggestions about what such a framework might look like but that is another story.

## Conflict of interest statement

The author declares that the research was conducted in the absence of any commercial or financial relationships that could be construed as a potential conflict of interest.
